# Cancer of Unknown Primary in Adolescents and Young Adults: Clinicopathological Features, Prognostic Factors and Survival Outcomes

**DOI:** 10.1371/journal.pone.0154985

**Published:** 2016-05-12

**Authors:** Kanwal Raghav, Hemendra Mhadgut, Jennifer L. McQuade, Xiudong Lei, Alicia Ross, Aurelio Matamoros, Huamin Wang, Michael J. Overman, Gauri R. Varadhachary

**Affiliations:** 1 Department of Gastrointestinal Medical Oncology, The University of Texas M.D. Anderson Cancer Center, Houston, Texas, United States of America; 2 Division of Cancer Medicine, The University of Texas M.D. Anderson Cancer Center, Houston, Texas, United States of America; 3 Department of Biostatistics, The University of Texas M.D. Anderson Cancer Center, Houston, Texas, United States of America; 4 Diagnostic Radiology, The University of Texas M.D. Anderson Cancer Center, Houston, Texas, United States of America; 5 Department of Pathology, The University of Texas M.D. Anderson Cancer Center, Houston, Texas, United States of America; Heinrich-Heine-University and University Hospital Duesseldorf, GERMANY

## Abstract

**Background:**

Cancer in adolescents and young adults (AYAs) (15–39 years) is increasingly recognized as a distinct clinical and biological entity. Cancer of unknown primary (CUP), a disease traditionally presenting in older adults with a median age of 65 years, poses several challenges when diagnosed in AYA patients. This study describes clinicopathological features, outcomes and challenges in caring for AYA-CUP patients.

**Methods:**

A retrospective review of 47 AYAs diagnosed with CUP at MD Anderson Cancer Center (6/2006–6/2013) was performed. Patients with favorable CUP subsets treated as per site-specific recommendations were excluded. Demographics, imaging, pathology and treatment data was collected using a prospectively maintained CUP database. Kaplan-Meier product limit method and log-rank test were used to estimate and compare overall survival. The cox-proportional model was used for multivariate analyses.

**Results:**

Median age was 35 years (range 19–39). All patients underwent comprehensive workup. Adenocarcinoma was the predominant histology (70%). A median of 9 immunostains (range 2–29) were performed. The most common putative primary was biliary tract based on clinicopathological parameters as well as gene profiling. Patients presented with a median of 2 metastatic sites [lymph node (60%), lung (47%), liver (38%) and bone (34%)]. Most commonly used systemic chemotherapies included gemcitabine, fluorouracil, taxanes and platinum agents. Median overall survival for the entire cohort was 10.0 (95% confidence interval (CI): 6.7–15.4) months. On multivariate analyses, elevated lactate dehydrogenase (Hazard ratio (HR) 3.66; 95%CI 1.52–8.82; *P = 0*.*004*), ≥3 metastatic sites (HR 5.34; 95%CI 1.19–23.9; *P = 0*.*029*), and tissue of origin not tested (HR 3.4; 95%CI 1.44–8.06; *P = 0*.*005*) were associated with poor overall survival. Culine’s CUP prognostic model (lactate dehydrogenase, performance status, liver metastases) was validated in this cohort (median overall survival: good-risk 25.2 months vs. poor-risk 6.1 months).

**Conclusions:**

AYA-CUP is associated with a poor prognosis. In the current “-omics” era collaborative research efforts towards understanding tumor biology and therapeutic targets in AYA-CUP is an unmet need, necessary for improving outcomes in young CUP patients.

## Introduction

Cancer in adolescents and young adults (AYAs) (15–39 years) is increasingly recognized as a distinct clinical and biological entity [1, 2]. AYAs with cancers have historically received less attention than either children or older adults with cancers. Managing cancer in these patients comes with several challenges owing to their unique clinical, psychological and socioeconomic demands [1–3]. Additionally, participation of AYAs in clinical trials has been inadequate for many reasons resulting in a relative lack of progress in this vulnerable group of cancer patients [4, 5]. Initiatives to overcome these impediments and research dedicated to cancers in AYAs have been encouraged by the National Cancer Institute to improve our understanding of cancers in this age-group and patient outcomes [6].

Cancer of unknown primary (CUP) is a heterogeneous group of cancers, for which despite detailed evaluation, the anatomical site of origin remains obscure [7, 8]. This is typically, a disease of older individuals, with a median age of 67 years. Management of these cancers requires a thorough physical examination, focused imaging and pathologic review. Treatment is site-specific therapy based on a putative primary when informed; otherwise, empiric platinum based chemotherapy is employed [7]. Nevertheless, survival outcomes in CUP patients remain suboptimal with a 9–13 month median overall survival in good performance status patients in empiric phase 2 studies [9].

A diagnosis of CUP in an AYA patient, although a rare clinical scenario, can present a formidable encounter. The trauma associated with a rare cancer entity coupled with the uncertainty regarding prognosis causes anxiety, leads to second guessing of the diagnosis, makes patients to feel isolated from other cancer patients with known primary cancers, and adversely affects the quality of life and treatment of these patients. Prior studies have demonstrated that AYAs have worse survival compared to their older counterparts in a variety of cancers, including colon and breast cancer; however the outcomes of AYAs with CUP has not been reported [1, 10]. Therefore, we performed this retrospective analysis of AYAs with CUP with the purpose of describing the clinicopathological profile and survival outcomes of CUP in AYAs, and delineating prognostic factors.

## Materials and Methods

We retrospectively reviewed data from 47 AYA patients who were evaluated and treated for CUP at The University of Texas M.D. Anderson Cancer Center, Houston, TX over a period of 7 years, between June 2006 and June 2013. The study was performed under a protocol approved by the institutional review board at M.D. Anderson Cancer Center and a waiver for informed consent was obtained. Patient records/information was anonymized and de-identified prior to analysis. For the purpose of this study, per ASCO guidelines, AYAs were defined as individuals between the ages of 15 and 39 years [1]. CUP was defined as presence of biopsy proven metastatic cancer without a detectable primary after a focused and comprehensive work-up including history and physical, laboratory assessment, pathology review including immunohistochemistry and diagnostic imaging with computerized tomography of chest, abdomen and pelvis [7]. Data on clinical presentation, demographics, imaging, pathology and treatment was collected from a prospectively maintained CUP database and the electronic medical record. Vital status was confirmed using tumor registry and patient records. Two clinicians independently reviewed each case and assigned a putative primary site based on the available clinicopathologic data. In cases where one of the physicians was involved with the care of the patient, the putative primary site was obtained from prior records and the second physician was blinded to this variable.

### Statistical methods

Patient and tumor characteristics including age, gender, ECOG performance status, number of metastatic sites (1, 2, 3+), site of metastases [lung, liver, bone, or lymph nodes], histology, laboratory parameters [lactic acid dehydrogenase (LDH) (≤ 618 IU/L, > 618 IU/L), albumin (≤ 4, > 4), circulating neutrophil-lymphocyte ratio (NLR) (< 5, ≥ 5)], immunohistochemistry [CK7, CK20, CDX2 (positive, negative or not performed)], first-line treatment, and use of molecular profiling tests to determine tissue of origin (ToO) were summarized using frequencies and percentages. Kaplan-Meier product limit method was used to estimate one-year overall survival and 95% confidence intervals (CI) and groups were compared using the log-rank test. Overall survival (OS) was measured from the date of diagnosis to the date of death or last follow-up for patients who were alive. Multivariate Cox proportional hazards models identified the factors predicting survival. Results were expressed in hazard ratios (HR) and 95% CI.

### Methods on concordance of primary site

We checked the agreement and disagreement of the provisional primary diagnoses submitted by each of the two clinicians and the putative primary stated by the ToO test. Cohen’s Kappa coefficient with 95% CI was used to assess the degree of agreement between two clinicians and ToO result. McNemar’s test was used to test the marginal homogeneity of the two clinicians agreeing with the results based on the ToO test; that is, whether they have the same tendency to agree or disagree with the results of ToO.

All tests were two-sided and *P-values* less than 0.05 were considered statistically significant. Statistical analyses were carried out using SAS 9.3 (SAS Institute Inc., Cary, NC), S-Plus 8.2 (TIBCO Software Inc.).

## Results

### Patient characteristics

Of the 714 CUP patients, 47 (6.5%) AYAs with CUP were included in the analyses. The baseline characteristics and key clinicopathologic features of this cohort are summarized in Table 1. The median age of diagnosis was 35 years (range: 19–39 years). Adenocarcinoma was the predominant histology (70%). Patients presented with a median of 2 metastatic sites, most common being lymph node (60%) followed by lung (47%), liver (38%) and bone (34%). Imaging including CT chest, abdomen and pelvis such as PET/CT scan, upper endoscopy, colonoscopy and mammography were performed in 60%, 51%, 47% and 40% patients, respectively. Most patients received systemic chemotherapy using gemcitabine, fluorouracil, taxanes and platinum agents, although a select few with solitary or oligometastatic disease were treated with radiation and surgery as primary modality therapy. Molecular profiling to determine the tissue of origin was performed in 26 (55.3%) patients with a positive prediction in 19 (73.0%) cases.

**Table 1:** Baseline patient characteristics and clinicopathological variables in 47 adolescents and young adult patients with cancer of unknown primary.

Variable	Patients N (%) (N = 47)	Variable	Patients N (%) (N = 47)
Age (years)		CDX2 immunostaining	
Median	35	Negative	14 (29.8%)
≤ 35	23 (48.9%)	Positive	12 (25.5%)
> 35	24 (51.1%)	Not done	21 (44.7%)
Gender		Neutrophil-lymphocyte ratio	
Female	30 (63.8%)	Low (≤ 5)	31 (70.6%)
Male	17 (36.2%)	High (> 5)	13 (29.4%)
Performance status (ECOG)		Lung metastasis	
0	11 (25.6%)	Absent	25 (53.2%)
1	20 (46.6%)	Present	22 (46.8%)
2	10 (23.2%)	Liver metastasis	
3	2 (4.6%)	Absent	29 (61.7%)
Number of metastatic sites		Present	18 (38.3%)
1	11 (23.4%)	Bone metastasis	
2	12 (25.5%)	Absent	31 (66.0%)
3+	24 (51.1%)	Present	16 (34.0%)
Histology		Lymph nodes metastasis	
Adenocarcinoma	33 (70.2%)	Absent	19 (40.4%)
Carcinoma	6 (12.7%)	Present	28 (59.6%)
Malignant neoplasm	4 (8.5%)	First line treatment	
Squamous cell carcinoma	2 (4.3%)	FOLFOX/CAPOX/5FU	11 (23.4%)
Other	2 (4.3%)	Platinum + Taxane	8 (17.0%)
Lactate dehydrogenase (IU/L)^**-**^		Gemcitabine based	8 (17.0%)
Normal (≤ 618)	25 (58.1%)	Other platinum based	6 (12.8%)
High (> 618)	18 (41.9%)	Radiation	4 (8.5%)
CK7 immunostaining		Surgery	4 (8.5%)
Negative	10 (21.3%)	Other therapy/Unknown	6 (12.8%)
Positive	28 (59.6%)	Tissue of origin performed	
Not done	9 (19.1%)	No	21 (44.7%)
CK20 immunostaining		Yes	26 (55.3%)
Negative	19 (40.4%)	Tissue of origin	
Positive	18 (38.3%)	Primary predicted	17 (65.4%)
Not done	10 (21.3%)	Primary not predicted	9 (34.6%)

### Univariate and multivariate survival analyses

**Fig 1:**
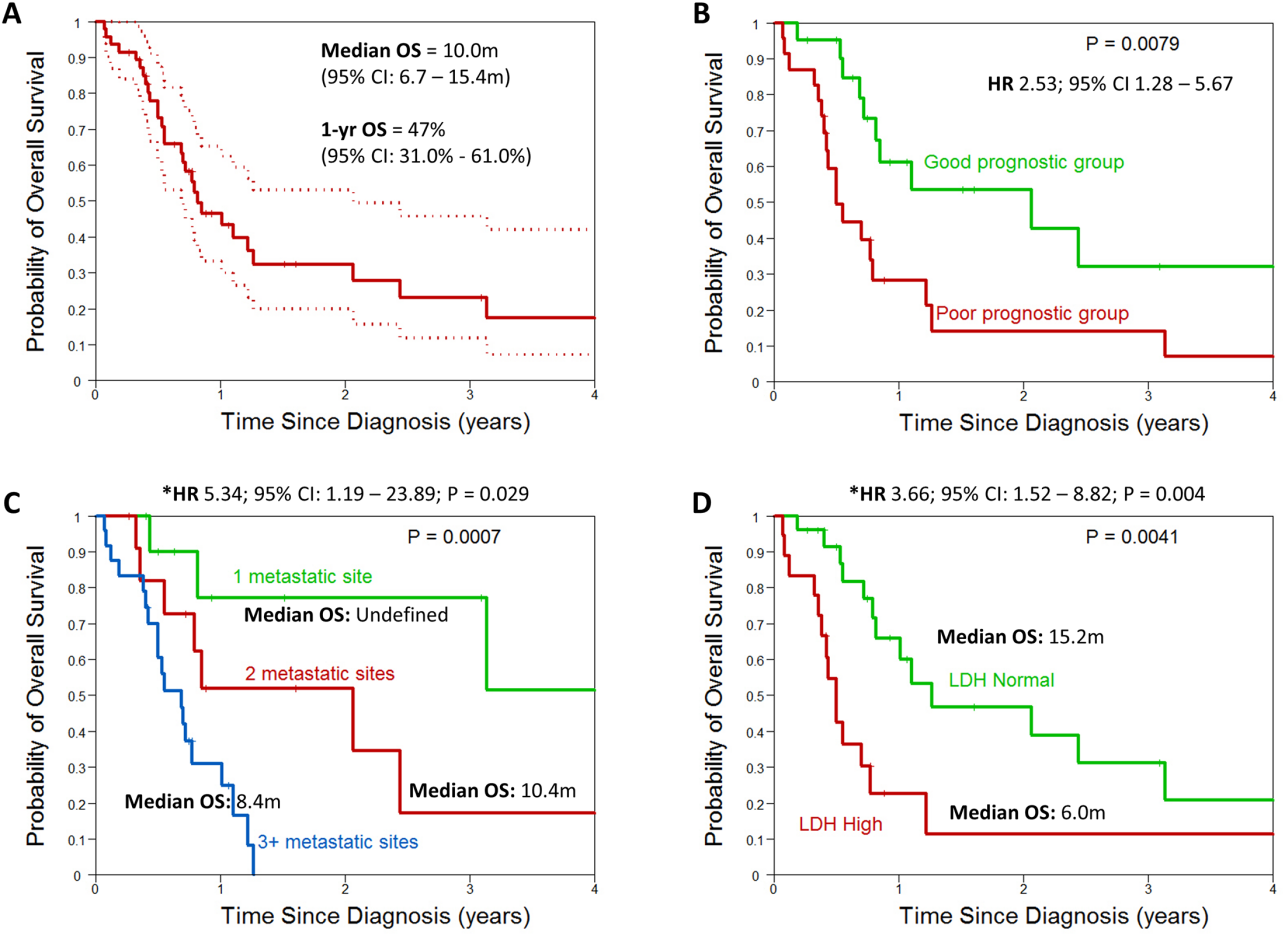
Kaplan-Meier curves of overall survival A) All patients, B) By Culine prognostic risk group, C) By number of metastatic sites and D) By lactate dehydrogenase levels.

The median follow up for the entire cohort was 8.6 months (range: 1–55 months) and at the time of the analysis, 30 (63.8%) patients had died. The 1-year OS for all patients was 47.0% (95% CI: 31.0%– 61.0%) and median OS of the entire cohort was 10.0 months (95% CI: 6.7–15.4 months) (Fig 1A).

On univariate analysis, number of metastatic sites (median OS for 1, 2, ≥3 sites: Undefined, 10.4 months, 8.4 months), presence of lung metastasis (median OS for metastasis 8.4 months vs. no metastasis 14.9 months), elevated lactate dehydrogenase (median OS for elevated LDH 6.1 months vs. normal 15.4 months), and tissue of origin tested (median OS for ToO not tested 8.6 months vs. ToO tested 15.4 months) significantly impacted OS (Fig 1C and 1D) (Table 2). On multivariate analysis, elevated lactate dehydrogenase (HR 3.66; 95%CI 1.52–8.82; *P = 0*.*004*), 3 or more metastatic sites (HR 5.34; 95%CI 1.19–23.9; *P = 0*.*029*) compared to 1 site, and tissue of origin not tested (HR 3.4; 95%CI 1.44–8.06; *P = 0*.*005*) were associated with worse survival (Table 2).

**Table 2:** Univariate Analysis of One-year Survival Estimates by Patient and Clinical Characteristics.

Variables	Patients	Events	Median Survival (Month)	1-Year Overall Survival Estimate (95% CI)	P Value
All	47	30	9.8	0.47 (0.31, 0.61)	
Age					
≤ 35	23	14	13.2	0.50 (0.27, 0.69)	
> 35	24	16	8.4	0.44 (0.23, 0.63)	0.43
Gender					
Female	30	20	9.8	0.48 (0.29, 0.65)	
Male	17	10	10.2	0.45 (0.20, 0.67)	0.46
Performance status (ECOG)					
0	11	4	24.8	0.68 (0.31, 0.89)	
1–3	32	24	9.5	0.38 (0.21, 0.55)	0.12
Number of metastatic sites					
1	11	3	NR	0.78 (0.36, 0.94)	
2	12	8	24.8	0.52 (0.21, 0.77)	
3+	24	19	8.2	0.32 (0.14, 0.51)	0.0007
Lactate dehydrogenase					
Normal	25	14	15.2	0.67 (0.44, 0.83)	
High	18	14	6.0	0.21 (0.06, 0.43)	0.004
Neutrophil-lymphocyte ratio					
≤ 5	31	18	15.2	0.59 (0.39, 0.75)	
> 5	13	10	8.4	0.25 (0.06, 0.50)	0.05
Lung metastasis					
No	25	13	14.7	0.64 (0.40, 0.80)	
Yes	22	17	8.2	0.28 (0.11, 0.49)	0.016
Liver metastasis					
No	29	18	12.1	0.56 (0.35, 0.73)	
Yes	18	12	6.0	0.33 (0.13, 0.55)	0.13
Bone metastasis					
No	31	18	10.2	0.52 (0.32, 0.69)	
Yes	16	12	8.3	0.38 (0.15, 0.61)	0.10
Lymph nodes metastasis					
No	19	11	13.2	0.52 (0.26, 0.72)	
Yes	28	19	9.3	0.44 (0.24, 0.62)	0.48
First line treatment					
5FU based	11	8	10.2	0.43 (0.14, 0.69)	
Gemcitabine based	8	3	15.2	0.83 (0.27, 0.97)	
Platinum based	14	12	6.0	0.15 (0.02, 0.39)	
Surgery + Radiation	8	3	37.7	0.85 (0.30, 0.98)	0.0005
Tissue of origin tested					
No	21	16	8.4	0.32 (0.13, 0.52)	
Yes	26	14	15.2	0.60 (0.37, 0.77)	0.007

### Validation of Culine prognostic model

Patients were risk stratified using the Culine prognostic model for CUP and classified as good risk (ECOG performance status of 0 or 1 and normal LDH or no evidence of liver metastases if LDH unknown) and poor risk (ECOG performance status of 2 or more or elevated LDH or presence of liver metastases if LDH unknown) [11]. Median survival of good risk patients was 25.2 months compared to 6.1 months for poor risk patients (HR 2.53; 95% CI 1.28–5.67; *P = 0*.*008*) (Fig 1B).

### Methods on concordance of primary site

Molecular tumor profiling predicted a tissue of origin in 19 of 26 (73.0%) cases; most common being biliary tract 5 (26.3%), ovarian 4 (21.0%) and gastroesophageal 2 (10.5%) primaries. The two clinicians were in agreement with regards to the putative primary in 33 of 47 cases (70.2%) and showed strong concordance (coefficient 0.89 [95% CI: 0.70–1.0]) amongst them. Of the available 19 cases where the putative primary was known based on the results from ToO test, the two clinicians individually agreed with these diagnoses in 9 (47.4%) and 10 (52.6%) cases showing fair concordance (coefficient 0.36 [95% CI 0.1–0.5] and 0.44 [95% CI 0.2–0.7]), respectively. The McNemar’s test revealed a *P value* of 0.37, indicating that the two clinicians had the same propensity to either agree or disagree with the results based on ToO test.

### Case illustrations

**Fig 2:**
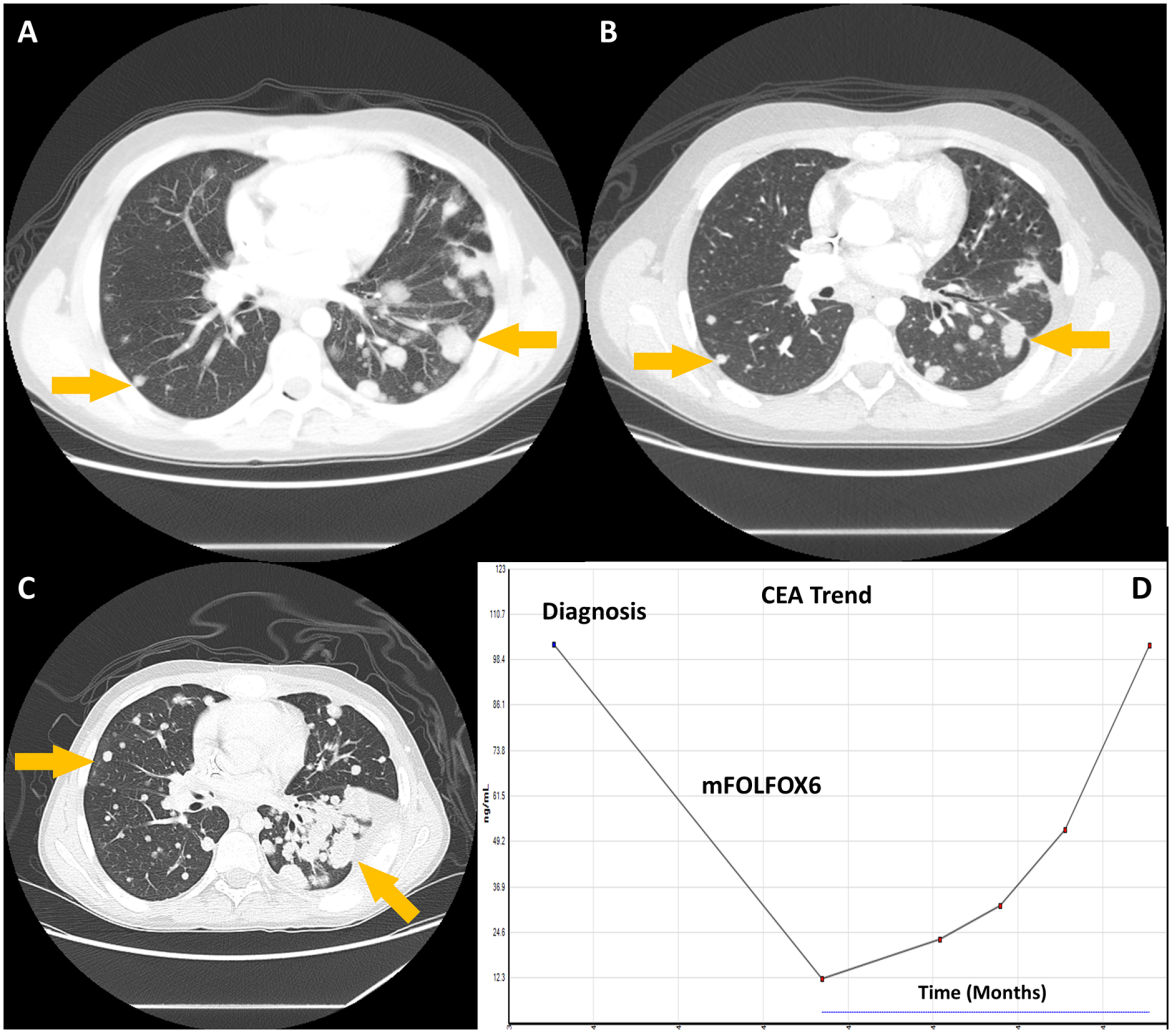
Case illustrating cancer of unknown primary with an intestinal profile. **A)** CT Chest at diagnosis shows multiple pulmonary metastases, **B)** CT Chest after 12 cycles of chemotherapy with 5-Fluorouracil and oxaliplatin showing radiographic response, C) CT Chest after 4 cycles of 5-Fluorouracil and irinotecan and clinical trial involving MEK1-2 inhibitor with progression of disease, **D)** Trend in tumor marker, carcinoembryonic antigen (CEA).

**Case 1:** A 17-year old previously healthy young man, without any significant family history of cancer, presented with progressive shortness of breath, cough, weight loss and back pain for past 3 months. Blood tests showed markedly elevated CA19-9 and CEA and normal Alpha feto-protein, Beta-HCG and chromogranin-A. CT imaging revealed too-numerous-to count bilateral lung metastases, mediastinal lymphadenopathy, and diffuses bony metastases (Fig 2A). Testicular ultrasound was normal. Lung biopsy revealed a poorly differentiated adenocarcinoma with immunohistochemistry (IHC) positive for CEA, CK20, CDX2, and negative for CK7, TTF-1, Napsin A, synaptophysin, and chromogranin. Upper endoscopy, colonoscopy and MR enterography done at an outside institution were negative for a primary cancer. Given the intestinal IHC profile, he was started on a colon specific regimen with 5FU and oxaliplatin (mFOLFOX6) to which bevacizumab was added after several cycles. He responded well to this regimen with clinical benefit noted in his cough and pain symptoms and decrease in CEA and CA19-9 (Fig 2D). Repeat CT imaging after 12 cycles of chemotherapy showed excellent partial response and he was on maintenance 5FU after oxaliplatin hypersensitivity (Fig 2B). On progression, patient was treated with additional colon cancer based regimens including 5FU and irinotecan with mild progression. Insufficient tissue prompted a repeat biopsy for biomarkers to help guide further lines of therapy. A next generation sequencing (NGS)-based analysis for the detection of somatic mutations in the coding sequence of a total of 50 genes was performed on the DNA extracted from the sample and showed mutations in SMAD4 and NRAS. Tumor was microsatellite stable. Cetuximab was not used and an attempt at empiric taxane based therapy and protocol-based MEK1-2 inhibitor showed no response with worsening disease (Fig 2C). He died 18 months after his initial presentation.

**Fig 3:**
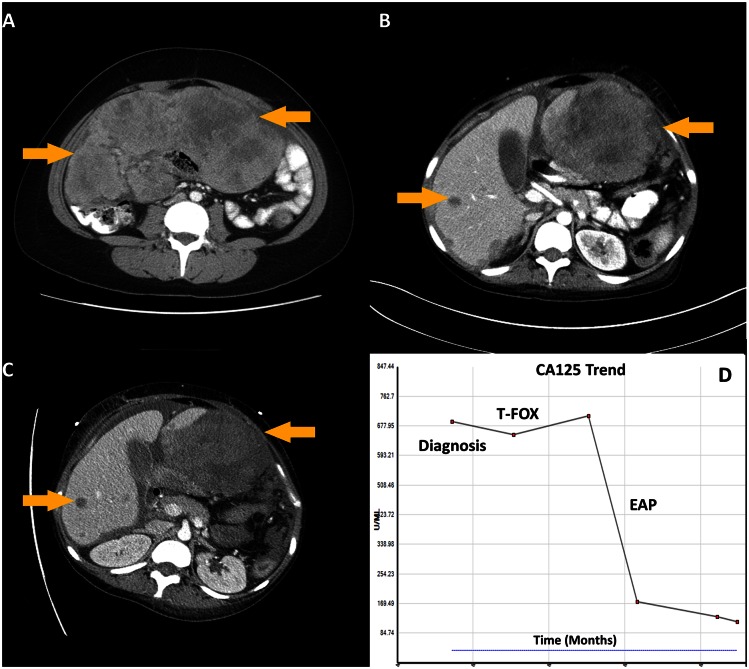
Case illustrating use of tissue of origin profiling in poorly differentiated neoplasms. **A)** CT Abdomen at diagnosis shows a large intrabdominal mass, **B)** CT Abdomen after 3 cycles of chemotherapy with 5-Fluorouracil paclitaxel, and oxaliplatin (T-FOX) shows progressive disease, **C)** CT Abdomen after 3 cycles of etoposide, adriamycin and ciplatin (EAP) shows radiographic response **D)** Trend in tumor marker, CA-125.

**Case 2:** A 24 year-old previously healthy young woman presented with a 2 month history of abdominal discomfort and bloating. Imaging revealed a 21 cm upper abdominal mass and peritoneal carcinomatosis (Fig 3A). Biopsy of the abdominal mass revealed a malignancy with epithelioid differentiation, favoring poorly differentiated adenocarcinoma with signet ring cells. Immunohistochemistry (IHC) was nonspecific and inconsistent (positive for pancytokeratin, PAX8, and CDX2, and negative for CK7, CK20, ER, WT1, calretinin, CD31, CD34, and synaptophysin) and working diagnosis was a putative upper gastrointestinal versus mullerian cancer profile. Blood tests showed elevated CA-125 and normal CEA and CA 19–9. EGD was negative and colonoscopy was incomplete secondary to extrinsic compression. Given the lack of clarity, tissue of origin testing was sent and in the interim she was started on empiric paclitaxel, 5-FU and oxaliplatin (T-FOX) for broad coverage of both upper GI and ovarian malignancies. She had progression of disease after 3 cycles (Fig 3B). At this point, tissue of origin testing results returned with a 90% likelihood of adrenocortical origin. Pathology was re-reviewed and immunohistochemical markers for adrenocortical carcinoma (ACC), including inhibin, calretinin, and melan A were negative and adrenals were normal on imaging. 50 gene mutation panel revealed no actionable mutations. She was initiated on etoposide, adriamycin and ciplatin (EAP) given activity in ACC as well as broad activity. She had response on both imaging and tumor markers after 3 cycles (Fig 3C and 3D) but unfortunately, subsequently had clinical decline and passed away approximately 6 months after her initial diagnosis. This case illustrates the discrepancy between ToO results and IHC and the challenges in managing AYA CUP patients with complex presentations.

## Discussion

This AYA-CUP study brings to attention a few important conclusions. First, in this cohort of 47 AYA –CUP patients, median survival was about 10.0 months with 53% of patients dying within the first year of diagnosis. This is similar to unselected older CUP patients where the median survival ranges from 7–12 month [11–13]. Second, traditional prognostic factors in CUP, such as the number of metastatic sites and elevated lactate dehydrogenase levels were independently associated with poor survival which validates the Culine prognostic model in AYA-CUP [11]. Notably, most patients seen at our institution had an evaluation done at outside institutions and it ranged widely from a minimalistic approach to excessive [14]. The immunophenotypic markers used to characterize these tumors was also varied. Despite guidelines there clearly exists a discrepancy in work-up with IHC and imaging for this heterogeneous disease [14].

Similar to results of large prospective trial of molecular tumor profiling to determine tissue of origin in older patients with CUP, molecular tumor profiling on this cohort of AYA patients predicted biliary tract to be the most common putative site of origin [9]. We showed that ToO results were compatible with the presumed primary diagnosis based on clinicopathologic findings in fewer cases than shown in prior studies [15]. Molecular profiling has been utilized in CUP based on validation studies performed in metastatic cancers of known primaries [15–17]. Recent studies suggest ToO can provide a specific lineage diagnosis in many patients with poorly-differentiated neoplasms unclassifiable by standard pathologic evaluation, and has the potential to complement standard pathologic evaluation when IHC is inconclusive [18, 19]. In the absence of additional well designed prospective validation and outcomes-based research, the current consensus guidelines do not recommend gene expression profiling in the standard management of CUP [20]. However, since studies indicate that there is a potential role of ToO testing in a subset of CUP; further studies are needed to assess the benefit of molecular assays in improving clinical outcomes in these CUP subsets.

Exploratory analysis showed patients undergoing definitive surgery or radiation had a quite robust median survival of 38.2 months compared to a survival of 15.4 months, 10.4 months and 6.1 months in patients receiving systemic chemotherapy with gemcitabine, fluorouracil and platinum/taxane-based regimens, respectively. The small sample size restricts evaluation of CUP subsets (e.g. those patients who present with nodal disease alone vs. osseous dominant vs. isolated carcinomatosis) and impact of specific chemotherapy doublets. Collaborative mutli-instituional databases are required to overcome this limitation in the study of CUP in AYAs.

The impact of ToO testing on survival outcomes in the AYA-CUP cohort in interesting but cannot be overemphasized. Patients who were tested did better than those who did not undergo testing and this likely represents a selection bias, such that a patient with poor expected outcome is unlikely to get molecular profiling.

Figs 1 and 2 describe patient cases that are typical for a CUP clinic. There are several questions that emerge e.g. in a patient with intestinal profile CUP, how best to interpret RAS testing? Patient in Fig 1 had IHC specific for lower gastrointestinal cancer (without a primary) with responses to the armamentarium of drugs used in colon cancer. Given the NRAS mutation, an EGFR inhibitor was not used. The case in Fig 2 describes the challenges of managing poorly differentiated neoplasms including non-specificity of IHC, discrepancy in IHC and ToO results and discussing this with young patients and their parents, and the ambiguity involved in the management of these very complex presentations.

Although this is the first study on this rare subset of patients, the small size of the cohort is a limitation and findings need corroboration in larger cohorts. Our study, as any other retrospective series, is also associated with certain drawbacks inherent to the nature of analyses, including selection, reporting (including missing data) and referral bias. Furthermore, the diverse diagnostic and treatment approaches, outside of our institution, can potentially confound these results. It is also important to note that favorable CUP subsets such as women with isolated adenocarcinoma involving the axillary lymph nodes, women with papillary serous adenocarcinoma of an unknown primary, squamous carcinoma of the cervical lymph nodes, undifferentiated or low grade neuro-endocrine carcinoma of unknown origin, poorly differentiated carcinoma of the midline concerning for extragonal presentation were excluded from this cohort due to referral patterns as these are best treated by site-specific recommendations. Therefore this study chiefly addresses the unfavorable subset. Nevertheless, this study is a first step highlighting the unmet need of investigations in this population.

Although, preliminary data has started to emerge regarding molecular profiling in CUP it is currently insufficient to envision personalization of therapy or introduction of targeted therapy [7, 21–23]. There is an urgent need to understand the role of “-omics” in CUP cancers and differentiate the biological dissimilarities between CUP in AYAs and adults. Methodical mutational analysis in various AYA-CUP subsets will help with understanding the biology and push the therapeutic envelope and allow these patients on biomarker matched clinical trials. Participation of AYAs with CUP in clinical trials should be encouraged to avoid prior experience and adverse impacts of low clinical trial accrual in this group [24, 25]. At this time whether patients will benefit from histology agnostic molecular-driven trials, such as the National Institute of Health MATCH study, remains to be seen, but we must offer our AYA-CUP patients the opportunity to enroll in these studies. Finally, as novel therapies are developed for known cancers, they will likely impact appropriate CUP subtypes. Over the next several years, we need to focus our efforts in refining AYA-CUP subsets and leverage selective genomics and proteomics techniques to eventually deliver validated new therapeutic approaches to our AYA-CUP patients.

In conclusion, CUP in AYAs is a challenging clinical entity associated with a morbid prognosis. Concerted research efforts are needed to tackle this orphan disease in this complex population.
